# Fine-Tuning BERT Models to Classify Misinformation on Garlic and COVID-19 on Twitter

**DOI:** 10.3390/ijerph19095126

**Published:** 2022-04-22

**Authors:** Myeong Gyu Kim, Minjung Kim, Jae Hyun Kim, Kyungim Kim

**Affiliations:** 1 College of Pharmacy, Graduate School of Pharmaceutical Sciences, Ewha Womans University, Seoul 03760, Korea; 2 College of Pharmacy, Yonsei University, Incheon 21983, Korea; minjung__kim@yonsei.ac.kr; 3 School of Pharmacy, Jeonbuk National University, Jeonju 54896, Korea; kimkimjh@jbnu.ac.kr; 4 College of Pharmacy, Korea University, Sejong 30019, Korea; kim_ki@korea.ac.kr

**Keywords:** bidirectional encoder representations from transformers (BERT), COVID-19, garlic, misinformation, Twitter

## Abstract

Garlic-related misinformation is prevalent whenever a virus outbreak occurs. With the outbreak of COVID-19, garlic-related misinformation is spreading through social media, including Twitter. Bidirectional Encoder Representations from Transformers (BERT) can be used to classify misinformation from a vast number of tweets. This study aimed to apply the BERT model for classifying misinformation on garlic and COVID-19 on Twitter, using 5929 original tweets mentioning garlic and COVID-19 (4151 for fine-tuning, 1778 for test). Tweets were manually labeled as ‘misinformation’ and ‘other.’ We fine-tuned five BERT models (BERT_BASE_, BERT_LARGE_, BERTweet-base, BERTweet-COVID-19, and BERTweet-large) using a general COVID-19 rumor dataset or a garlic-specific dataset. Accuracy and F1 score were calculated to evaluate the performance of the models. The BERT models fine-tuned with the COVID-19 rumor dataset showed poor performance, with maximum accuracy of 0.647. BERT models fine-tuned with the garlic-specific dataset showed better performance. BERTweet models achieved accuracy of 0.897–0.911, while BERT_BASE_ and BERT_LARGE_ achieved accuracy of 0.887–0.897. BERTweet-large showed the best performance with maximum accuracy of 0.911 and an F1 score of 0.894. Thus, BERT models showed good performance in classifying misinformation. The results of our study will help detect misinformation related to garlic and COVID-19 on Twitter.

## 1. Introduction

Misinformation is false or inaccurate information that is communicated intentionally or unintentionally [1]. Misinformation related to disease prevention and treatment could result in decisions that are harmful to health. Since the outbreak of coronavirus disease 2019 (COVID-19), misinformation on COVID-19 has been spreading rapidly [2], including the use of ultraviolet lamps, bathing in hot water, and spraying bleach into the body to prevent infection. Many researchers are interested in detecting vaccines or drug-related misinformation that directly affect public health. Dietary supplements such as garlic, pepper, chili, black seeds, honey, onion, vitamins D and C, and zinc have also been major topics of misinformation [3,4]. People tend to take dietary supplements without scientific evidence. However, blind faith in ineffective supplements can increase the risk of COVID-19 infection. Garlic, in particular, has been suggested as a treatment whenever viral diseases such as Ebola and severe acute respiratory syndrome-associated coronavirus (SARS-CoV) have occurred.

Social media sites such as Twitter are leading sources of misinformation about COVID-19 [5]. Misinformation spreads faster than accurate information through social media—faster than the virus itself [6]. The World Health Organization (WHO) created and distributed ‘Mythbusters’ to social media to prevent misinformation from spreading [7]. Although some researchers have suggested that garlic is effective in treating COVID-19, it is not universally accepted. Public institutions regard garlic-related content as misinformation. The WHO account tweeted a garlic-related mythbuster in February 2020 that there is no evidence that eating garlic has protected people from COVID-19. Nevertheless, garlic-related misinformation is still prevalent on Twitter and must be analyzed carefully.

Studies using social media posts as a dataset have been actively conducted [8]. However, it is difficult to identify misinformation among the vast number of social media posts. Researchers have conducted studies to automatically detect COVID-19 misinformation using natural language processing (NLP). Most recently, pretrained language models have proved useful in learning common language representations by utilizing a large amount of unlabeled data. Bidirectional Encoder Representations from Transformers (BERT), which was developed by researchers at Google and a product of Google, is designed to pretrain deep bidirectional representations from unlabeled text for masked word prediction and next-sentence prediction tasks [9]. After training, the pretrained representations are applied to many downstream NLP tasks by fine-tuning the pretrained parameters [9]. This fine-tuning is relatively inexpensive compared to pretraining and has achieved outstanding results in many tasks, including text classification [9,10]. Ayoub et al. fine-tuned a distilled version of BERT (DistilBERT) model using COVID-19 claims from several sources (e.g., WHO, Centers for Disease Control and Prevention, Cable News Network, and FactCheck) [11]. The model was tested with a subset of “CONSTRAINT shared task-2021 dataset” (5100 fake news and 5600 true news data) [12] and demonstrated high accuracy of 0.938 [11]. Using the “CONSTRAINT shared task-2021 dataset”, Birader et al. classified posts as ‘Fake’ or ‘Real’ and found that BERT performed better than other context-based embeddings such as XLNet and Embeddings from Language Model (ELMo) [13]. A single BERT classifier showed accuracy of 0.97 and an F1 score of 0.97 [13]. Qasim et al. fine-tuned nine BERT-based models using a “COVID-19 fake news dataset” (10,202 news posts gathered from Facebook, Instagram, various websites, and Twitter blogs) [14]. All classifiers showed excellent performance, and the Robustly optimized BERT approach (RoBERTa)-base model achieved the highest accuracy of 0.997 for classifying COVID-19 fake news [14]. However, the contents related to garlic in the datasets used in these studies are sparse. Hence, since these models were not fine-tuned with data on garlic, it is difficult to apply them for the detection of garlic-related misinformation.

Garlic-related misinformation was studied together with other misinformation (e.g., salt gargling, sarin gas, mosquito-borne disease, and 5th generation mobile network technology) in an Arabic tweet study [15]. The researchers employed a combination of several NLP methods (term frequency-inverse document frequency [TF-IDF], Word2Vec, and FastText) and machine learning classifiers (naïve Bayes, support vector machine, random forest, Extreme Gradient Boosting, Stochastic Gradient Descent, convolutional neural network, recurrent neural network, and convolutional recurrent neural network) [15]. The model with the best performance showed accuracy of 0.87 [15]. However, a low recall of 0.27 and an F1 score of 0.39 indicated low sensitivity of the model; moreover, the researchers did not apply BERT.

Therefore, this study aimed to apply the BERT model for classifying misinformation on garlic and COVID-19 on Twitter. In addition, we compared fine-tuning of these BERT models using our garlic-specific dataset with a general COVID-19 rumor dataset.

## 2. Materials and Methods

### 2.1. Study Design

**Figure 1:**
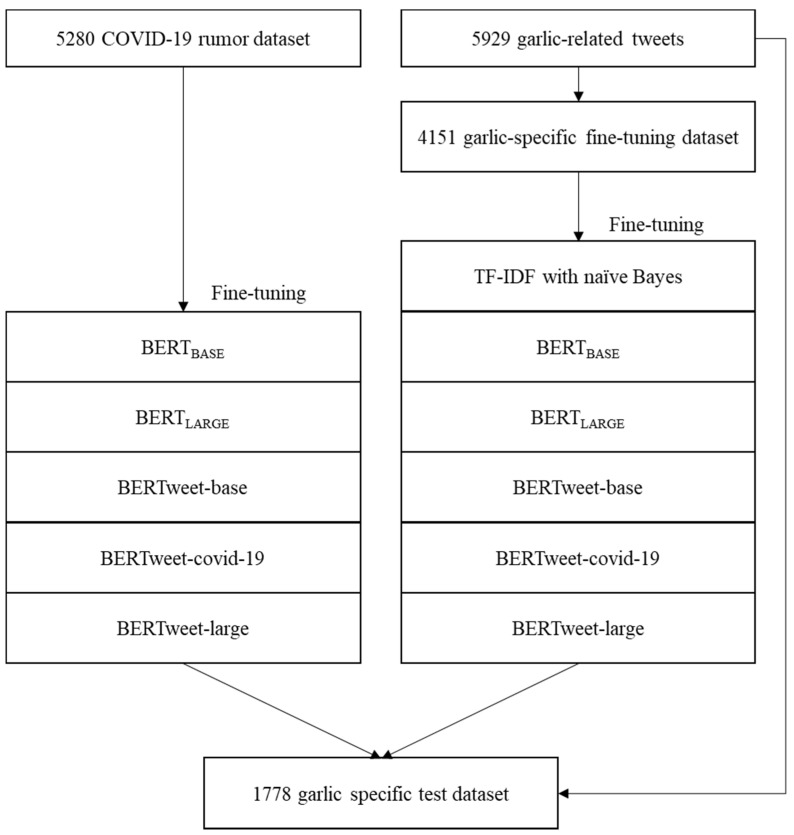
Study flow diagram.

This study was designed to fine-tune BERT models with a “COVID-19 rumor dataset” or a garlic-specific dataset and classify garlic-related COVID-19 misinformation from tweets. Figure 1 shows the study flow diagram. The study was exempted from Institutional Review Board review (202004-HR-012-01).

### 2.2. COVID-19 Rumor Dataset

Since the “CONSTRAINT shared task-2021 dataset” has only 0.19% of all garlic-related posts, the “COVID-19 rumor dataset” with more garlic-related posts (0.51%) was selected. The “COVID-19 rumor dataset” contains manually labeled 6834 data points (4129 sentences from news posts and 2705 sentences from tweets) [16]. The veracity assessment of a sentence can be true (the content is logical and describing facts), false (the content is made up or contains false information), or unverified (the authenticity or truthfulness of the statement is difficult to judge at the time of labeling) [16]. All data are freely available on GitHub (https://github.com/MickeysClubhouse/COVID-19-rumor-dataset, accessed on 19 February 2022). We used only 1699 true and 3581 false sentences.

### 2.3. Garlic-Specific Dataset and Labeling

From November 2019 to April 2020, 17,711 tweets mentioning both garlic and COVID-19 were collected. The search query was ‘(covid OR corona) AND garlic’. Python version 3.7 (Python Software Foundation, Fredericksburg, VA, USA) and Twitter premium application programming interface were used. Of 17,711 tweets, 5929 tweets written in English were selected after removing retweets, which were then randomly divided into fine-tuning and test datasets consisting of 70% (*n* = 4151) and 30% (*n* = 1778) of the tweets, respectively.

Two annotators independently classified the tweets into ‘misinformation’ (label = 1) and ‘other’ (label = 0) based on the tweet text. Based on WHO’s mythbusters, ‘misinformation’ was defined as tweets stating that garlic is effective in preventing or treating COVID-19. ‘Other’ included tweets delivering true information, sarcasm, and irrelevant information. Any discrepancy was resolved by discussion and, if required, consulting a third party. The agreement between the two annotators had a Cohen’s kappa value of 0.98. There was no significant difference in the distribution of misinformation between the fine-tuning and test datasets (43.7% and 43.5%, respectively; *p* = 0.887).

### 2.4. Data Preprocessing

Handles (in the form of ‘@name’), Uniform Resource Locator (URL), white spaces, and nonASCII words and characters were removed from the text of both the COVID-19 rumor and garlic-specific datasets. We inserted a space between punctuation marks and converted a word to lower case.

### 2.5. TF-IDF Vectorization with Naïve Bayes Classification

TF-IDF is an important measure that reflects the importance of a word. TF is a part of the term-weighting system measuring the frequency of occurrence of terms in the documents. Terms that are frequently mentioned in individual documents has a high TF. However, if the high frequency term is not concentrated on a particular document and is prevalent in the entire document, the performance of classification of documents using the term is poor. IDF is the logarithmically scaled inverse fraction of the documents that contain the term. TF-IDF is obtained by using the product of the TF and IDF (TF × IDF) [17]. A term that is specific to a document has high TF-IDF, while a term that occurs in the whole document has low TF-IDF. We used sklearn’s ‘TfidfVectorizer’ to calculate TF-IDF values and obtained a maximum of 5000 features of N-grams (unigrams, bigrams, trigrams, unigrams + bigrams, and unigrams + bigrams + trigrams). The minimum document frequency was set to 2, English stop words were ignored, and sublinear tf scaling was used. Then, a multinomial Naïve Bayes classifier with 5-fold cross-validation was used for text classification with each TF-IDF N-gram vector.

### 2.6. Fine-Tune BERT for Text Classification

**Figure 2:**
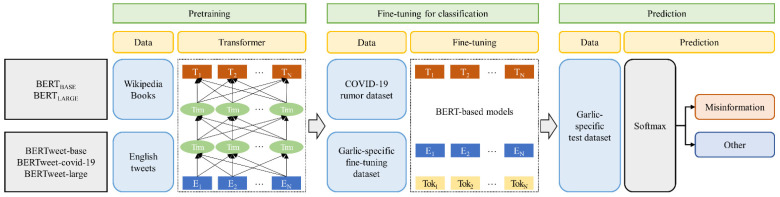
Pipeline of BERT-based models for classification task.

Figure 2 shows the whole pipeline of the BERT-based models for classification task (pretraining, fine-tuning, and prediction).

Table 1 shows the five BERT-based models (BERT_BASE_, BERT_LARGE_, BERTweet-base, BERTweet-COVID-19, and BERTweet-large) employed in this study. BERT uses the encoder stack of transformer model. BERT_BASE_ and BERT_LARGE_ have 12 and 24 layers in the Encoder stack, respectively [9]. BERTweet was trained based on the RoBERTa pretraining procedure [18]. The BERT models receive tokenized input and pass the input to the above layers. We used a tokenizer provided by each BERT model that splits input sentences into tokens and mapped the tokens to their IDs. All sentences were padded to a single, fixed length of 128 tokens. The BERT model uses an attention mask, which is a binary tensor indicating the position of the padded indices. The output of the hidden vectors is given into a softmax layer for NLP tasks. BERT was pretrained using English Wikipedia and BookCorpus while BERTweet was pretrained using English tweets [9,18].

The fine-tuning dataset was divided into training (80%) and validation (20%) data points. For classification task, we attached a classification block on top of BERT-based models. We used ‘BertForSequenceClassification’ and ‘RobertaForSequenceClassification’ interfaces designed for the classification task. These are normal BERT models with an added single-linear layer on top for classification that will be used as a sentence classifier. As we feed input data, the entire pretrained BERT model and the additional untrained classification layer is trained on our specific task. For the purpose of fine-tuning, we set up hyperparameters: learning rate for Adam optimizer = 2 × 10^−5^; number of epochs = 8; batch size = 32.

### 2.7. Statistical Analysis

The predicted class was obtained by applying the classifier to the test dataset. We created a 2 × 2 confusion matrix with true and predicted classes. The accuracy and F1 score (harmonic mean of precision and recall) were calculated as follows:

Accuracy = (TP + TN)/(TP + TN + FP + FN)

Precision = TP/(TP + FP)

Recall = TP/(FN + TP)

F1 score = (2 × Precision × Recall)/(Precision + Recall)

where TP, TN, FP, and FN mean true positive, true negative, false positive, and false negative, respectively.

The F1 score is used to evaluate the classification performance for imbalanced data, and a high F1 score means high precision and high recall [19].

## 3. Results

**Figure 3:**
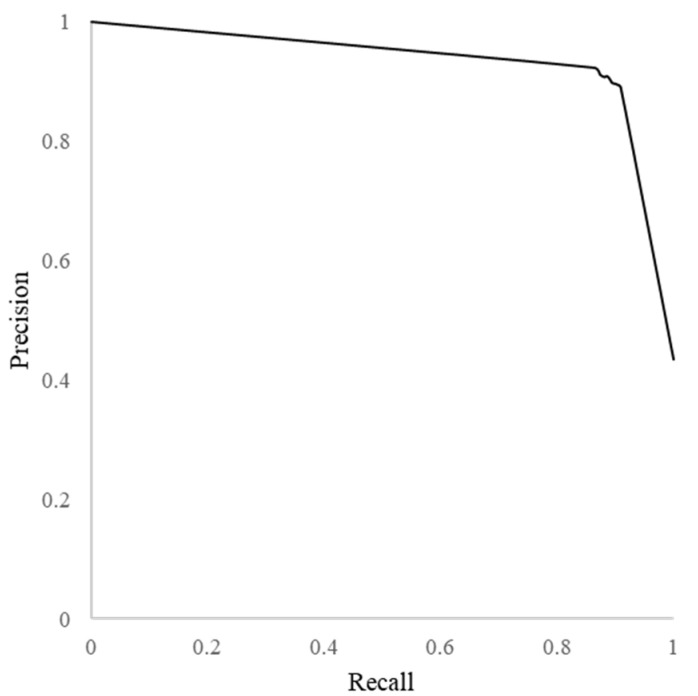
Precision-recall curve (BERTweet-large model).

Table 2 shows the classification performance of the BERT models. The BERT models fine-tuned with the COVID-19 rumor dataset showed poor performance, with maximum accuracy of 0.647. Using the garlic-specific dataset, traditional TF-IDF vectorization with naïve Bayes classification showed accuracy of 0.839 and an F1 score of 0.799. The performances using BERT models fined-tuned with the garlic-specific dataset were superior to those of BERT models fine-tuned with the COVID-19 rumor dataset and TF-IDF vectorization with naïve Bayes classification. The BERTweet models achieved accuracy of 0.897–0.911, while BERT_BASE_ and BERT_LARGE_ achieved accuracy of 0.887–0.897. BERTweet-large showed the best performance: the maximum accuracy was 0.911, and the F1 score was 0.894. Figure 3 shows a precision-recall curve for BERTweet-large model, indicating a high precision and recall.

**Figure 4:**
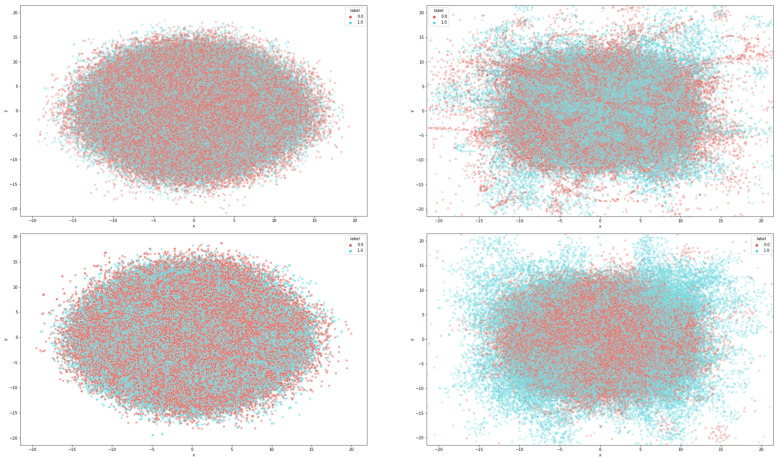
t-SNE visualization of the first hidden-layer embeddings (**upper left**) and the last hidden-layer embeddings (**upper right**) before fine-tuning and the first hidden-layer embeddings (**lower left**) and the last hidden-layer embeddings (**lower right**) after fine-tuning.

Figure 4 shows a t-Stochastic Nearest Neighbor (t-SNE) dimensionality reduction mapping comparing the hidden-layer embeddings before and after fine-tuning. This shows evident clustering through the fine-tuned BERTweet-large model.

Table 3 shows some examples of predicted results with the BERTweet-large model. There were 682, 939, 66, and 91 tweets as TP, TN, FP, and FN, respectively. The BERTweet-large model accurately classified some tweets delivering misinformation as ‘misinformation’ and true information, sarcasm, and irrelevant information as ‘other’.

## 4. Discussion

We have demonstrated that the fine-tuned BERTweet-large model showed the best performance in classifying misinformation on garlic and COVID-19 on Twitter. The model showed higher accuracy and F1 score than a previous garlic study that used Word2Vec and FastText (accuracy of 0.87 and an F1 score of 0.39) [15]. In addition, the BERT models were superior to traditional TF-IDF vectorization in this study. BERT has advantages over TF-IDF vectorization and static word embedding (Word2Vec and FastText). TF-IDF, Word2Vec, and FastText are context-independent methods. Different senses of the word (e.g., Python has two different meanings: as a programming language or a genus of snakes) are combined into one single vector. However, the BERT model is context-dependent and generates embeddings that allow more than one vector representation for the same word, based on the context in which the word is used.

For fine-tuning BERT, the COVID-19 rumor dataset showed poorer performance than the garlic-specific dataset. The COVID-19 rumor dataset included 35 sentences (0.51%) containing garlic and they were all false information. Since none of the sentences related to garlic were classified as ‘other,’ BERT must not have been properly fine-tuned to classify tweets related to garlic. In previous studies, when the scope of the data used for fine-tuning was similar to the scope of the test data, the accuracy was greater than 0.9 [11,13,14]. However, our study showed that the general misinformation dataset may not be suitable for certain subjects, such as garlic.

The BERTweet models showed better performance than BERT_BASE_ and BERT_LARGE_. Tweets have different characteristics from Wikipedia or BooksCorpus used in pretraining BERT_BASE_ and BERT_LARGE_. Tweets are short and use irregular words such as abbreviations and typographical errors [18]. BERTweet was pretrained for English tweets and outperformed in various NLP tasks, including text classification [18]. In this study, BERTweet-large with 355M parameters showed the best performance. BERTweet-COVID-19, pretrained with COVID-19 tweets, showed better performance in classifying misinformation related to COVID-19 than the BERTweet-base model. The accuracy of BERTweet-COVID-19 exceeded 0.9, without any substantial difference from BERTweet-large. However, fine-tuning took less time with BERTweet-COVID-19 than BERTweet-large; indeed, fine-tuning of BERTweet-large took a longer time than all other BERTweet models.

Nevertheless, the accuracy of our garlic study was lower than that of previous, general COVID-19 misinformation studies [11,13,14]. This is because garlic was widely used as an element of sarcasm. BERTweet-large misclassified some sarcastic tweets into misinformation: “Just rubbing my crucifix with garlic before I head out for another COVID-free day” or “Anyone knows a good recipe to make with Garlic, Sunlight, Alcohol, Nasal spray and Chlorine? Want to become the next millionaire with a product to kill Corona. Credits: Whatsapp University.” These tweets were ambiguous, and only the context in each tweet was considered. Their perspective on garlic may have been expressed in pre- or post-tweets as Twitter threads. Thus, it seems difficult for BERT models to learn the connotation of text from such posts.

This study has several limitations. First, some search terms (e.g., ‘Allium sativum’ and ‘SARS-CoV-2′) were omitted. However, considering the length restriction and a high proportion of nonexperts on Twitter, users may use more simple words than long and professional words. Second, search queries, tweets, and BERTweet models were limited to English. However, as garlic is commonly used in non-English countries, tweets related to garlic are likely to be written in non-English characters. Therefore, not detecting such posts or including them in the dataset would have led to exclusion of important/relevant data. Finally, our model is useful for classifying garlic-related COVID-19 misinformation on Twitter. As BERTweet is a tweet-specific BERT model, it will be difficult to apply to other social media platforms.

## 5. Conclusions

Misinformation about dietary supplements, including garlic, is widespread due to the COVID-19 outbreak. The BERTweet-large model showed good performance in classifying misinformation on Twitter. The results of our study will help detect misinformation related to garlic and COVID-19 on Twitter.

## Figures and Tables

**Table 1:** BERT models used in the study.

Model	Number of Parameters	Pretraining Data
BERT_BASE_	110 M	2500 M words from English Wikipedia and 800 M words from BookCorpus
BERT_LARGE_	340 M	Same as BERT_BASE_
BERTweet-base	135 M	850 M English tweets
BERTweet-COVID-19	135 M	23 M COVID-19 English tweets
BERTweet-large	355 M	873 M English Tweets

**Table 2:** Classification performance of BERT models.

Model	Fine-Tuning Datasets			
	COVID-19 Rumor Dataset		Garlic-Specific Dataset	
	Accuracy	F1 Score	Accuracy	F1 Score
TF-IDF with naïve Bayes	-	-	0.839	0.799
BERT_BASE_	0.620	0.399	0.887	0.864
BERT_LARGE_	0.621	0.570	0.897	0.874
BERTweet-base	0.622	0.589	0.897	0.876
BERTweet-COVID-19	0.647	0.588	0.901	0.880
BERTweet-large	0.626	0.563	0.911 *	0.894 *

* Bolds represent the highest score.

**Table 3:** Predicted results with BERTweet-large model.

Category	N *	Type	Example Tweet (Paraphrased to Ensure Anonymity)
True positive (misinformation predicted as misinformation)	682		“Good news!!! Corona virus can be cured with a bowl of freshly boiled garlic water. An old Chinese doctor proved its effectiveness. Many patients have also confirmed that this is effective.”
True negative (other predicted as other)	939	True information	“COVID-19 Updates: While Garlic is a healthy food with some antimicrobial properties, there is no evidence that eating garlic has protected people from coronavirus.—WHO”
		Sarcasm	“To protect against the corona virus, eat two cloves of garlic and raw onion every morning and evening. Basically, it’s useless but will keep everyone at a safe distance.”
		Irrelevant information	“Corona Food Diaries Day 11: Kale and Pasta in a Garlic Parmesan white wine sauce topped with grilled chicken.”
False positive (other predicted as misinformation)	66		“We have ginger, turmeric, garlic, and black pepper in our diet regularly. They protect us from common cold, cough, sore throat, and lung mucus. But it is unknown whether they can provide protection against the corona virus.”
False negative (misinformation predicted as other)	91		“Corona Virus or COVID-19 does not resist ginger and garlic. Regions using much garlic and ginger have recorded insignificant number of cases of this outbreak.”

* Number of tweets predicted by category out of 1778 test data.

## Data Availability

The data presented in this study are available on request from the corresponding author. The data are not publicly available due to privacy.

## References

[1] Wu L., Morstatter F., Carley K.M., Liu H. (2019). Misinformation in social media: Definition, manipulation, and detection. ACM SIGKDD Explor. Newsl..

[2] Radu R. (2020). Fighting the ‘Infodemic’: Legal responses to COVID-19 disinformation. Soc. Media Soc..

[3] Adams K.K., Baker W.L., Sobieraj D.M. (2020). Myth busters: Dietary supplements and COVID-19. Ann. Pharm..

[4] Alotiby A. (2021). The impact of media on public health awareness concerning the use of natural remedies against the COVID-19 outbreak in Saudi Arabia. Int. J. Gen. Med..

[5] Abbasi-Kangevari M., Kolahi A.A., Ghamari S.H., Hassanian-Moghaddam H. (2021). Public knowledge, attitudes, and practices related to COVID-19 in Iran: Questionnaire study. JMIR Public Health Surveill..

[6] Radwan E., Radwan A., Radwan W. (2020). The role of social media in spreading panic among primary and secondary school students during the COVID-19 pandemic: An online questionnaire study from the Gaza Strip, Palestine. Heliyon.

[7] Coronavirus Disease (COVID-19) Advice for the Public: Mythbusters. https://www.who.int/emergencies/diseases/novel-coronavirus-2019/advice-for-public/myth-busters#garlic.2021.

[8] Lee J.Y., Lee Y.S., Kim D.H., Lee H.S., Yang B.R., Kim M.G. (2021). The use of social media in detecting drug safety-related new black box warnings, labeling changes, or withdrawals: Scoping review. JMIR Public Health Surveill..

[9] Devlin J., Chang M.W., Lee K., Toutanova K. BERT: Pre-training of deep bidirectional transformers for language understanding. Proceedings of the NAACL-HLT.

[10] Sun C., Qiu X., Xu Y., Huang X. How to fine-tune BERT for text classification?. Proceedings of the CCL: China National Conference on Chinese Computational Linguistics.

[11] Ayoub J., Yang X.J., Zhou F. (2021). Combat COVID-19 infodemic using explainable natural language processing models. Inf. Process Manag..

[12] Patwa P., Sharma S., Pykl S., Guptha V., Kumari G., Akhtar S., Ekbal A., Das A., Chakraborty T. Fighting an infodemic: COVID-19 fake news dataset. Proceedings of the Constraint 2021.

[13] Biradar S., Saumya S., Chauhan A. (2022). Combating the infodemic: COVID-19 induced fake news recognition in social media networks. Complex Intell. Syst..

[14] Qasim R., Bangyal W.H., Alqarni M.A., Ali Almazroi A. (2022). A fine-tuned BERT-based transfer learning approach for text classification. J. Healthc. Eng..

[15] Alqurashi S., Hamoui B., Alashaikh A., Alhindi A., Alanazi E. (2021). Eating garlic prevents COVID-19 infection: Detecting misinformation on the Arabic content of Twitter. arXiv.

[16] Cheng M., Wang S., Yan X., Yang T., Wang W., Huang Z., Xiao X., Nazarian S., Bogdan P. (2021). A COVID-19 rumor dataset. Front. Psychol..

[17] Salton G., Buckley C. (1988). Term-weighting approaches in automatic text retrieval. Inf. Process Manag..

[18] Nguyen D.Q., Vu T., Nguyen A.T. BERTweet: A pre-trained language model for English Tweets. Proceedings of the 2020 Conference on Empirical Methods in Natural Language Processing.

[19] Kim M.G., Kim J., Kim S.C., Jeong J. (2020). Twitter analysis of the nonmedical use and side effects of methylphenidate: Machine learning study. J. Med. Internet Res..

